# Pioneering the Use of Tracker Data to Evaluate Lean-Led Hospital Design

**DOI:** 10.1177/19375867231226440

**Published:** 2024-02-12

**Authors:** Hannelore Schouten, Stefan Heusinkveld, Jos Benders

**Affiliations:** 1 Department of Management and Organization, Vrije Universiteit, Amsterdam, the Netherlands; 2 Institute for Management Research, Radboud Universiteit, Nijmegen, the Netherlands; 3 Department of Industrial Economics and Technology Management, NTNU, Trondheim, Norway; 4 CESO, KU Leuven, Belgium

**Keywords:** Lean, Lean-led hospital design, postoccupancy evaluation, movement tracking, nursing staff

## Abstract

**Objective:**

This study aims to examine how we can effectively and affordably evaluate the impact of design concepts such as Lean-Led Hospital Design (LLHD) on the allocation of nurses’ time spent at different locations. Particularly in patient rooms, as this can be seen as value-adding time.

**Background:**

LLHD aims to create a hospital environment that supports value creation for patients and reduces waste. However, only a few studies measure its’ effects. One of the reasons for this absence is the lack of an adequate and affordable way to evaluate.

**Method:**

Nurses’ time spent in patient rooms was used as a proxy for value-adding time. Through studying a pioneering case of LLHD, and drawing on a pre-/postoccupancy evaluation approach, this study used an innovative methodology utilizing mobile tracking devices to adequately provide reliable data about the time nurses spend at specific locations.

**Results:**

Our analysis reveals that the answer to the question concerning the impact of LLHD, as advocated by its proponents, on nurses’ allocation of time for value-adding activities versus waste time remains inconclusive. Our findings indicate no discernible difference in the amount of value-adding time nurses spent in the old facility compared to the new one.

**Conclusion:**

Our experience suggests that mobile tracking devices offer an affordable, efficient means of collecting data that produces objective measurements. Nevertheless, the interpretation of this time-based data necessitates the inclusion of supplementary qualitative information.

## Pioneering Using Tracker Data

Healthcare organizations continuously seek methods to enhance the quality and efficiency of their care processes (e.g., Knudsen et al., 2019). Lean-inspired changes have been prominent among these methods for over two decades now. Lean thinking involves five key principles: identifying customer value, identifying the value stream, creating flow through waste elimination, introducing pull, and managing toward perfection (Womack & Jones, 1996). Applying these general ideas should lead to operational improvements. However, as Grunden and Hagood (2012) emphasize, this necessitates a specification of how these principles can be applied in a hospital setting. In their book *Lean-Led Hospital Design* (LLHD), they contend that the structured application of Lean thinking to hospital facility design might result in an environment that supports Lean working processes, thereby providing more value for patients and reducing wasted time.

To substantiate this claim, we got the unique opportunity to conduct a case study to gain insight into the impact of implementing LLHD on the value-adding time of nursing staff at Care-Face, a hospital that adopted Lean as its guiding principle for operations and used LLHD as the corner stone for designing its new hospital facility (Schouten et al., 2021). CareFac’s new facility, designed based on LLHD principles, was opened in January 2017. A prime objective of using LLHD was to increase the time nurses spent at patients’ bedsides while reducing time spent on non-value-adding activities such as walking around searching for equipment or medication. As per Graban’s (2011) delineation, the senior management team at CareFac unambiguously classified the time allocated by nurses at patients’ bedsides as “value-adding time” while categorizing extraneous movements and actions as “waste.” This strategic shift was driven by the experience of significant nonvalue-adding movement due to an outdated facility design in the old facility, which had become inefficient over time. Consequently, reducing unnecessary walking time became a central focus during the design of the new facility.

In Autumn 2016, just before moving to the new facility, the hospital board decided to evaluate these design principles and requested an affordable and rapid evaluation. These constraints necessitated an innovative approach, given the limited time available to set up the study. The first author assumed the role of a participatory action researcher, benefiting from her affiliation with both the hospital and the university. This approach enabled the collection of data both before and after moving to the new facility.

Despite a substantial body of literature developed over the past two decades regarding the successes and failures of Lean implementation processes in hospitals, as well as the adoption rate of specific Lean tools and methods (e.g., Benders, Van Grinsven, and Ingvaldsen, 2019a; Lima et al., 2021; Moraros et al., 2016; Scoville & Little, 2014; Shortell et al., 2018), there has been relatively little research on the effectiveness of Lean-based interventions (D’Andreamatteo et al., 2015; Lima et al., 2021; Moraros et al., 2016). Moraros et al. (2016, p. 163) assert: “In our pursuit to assess how we can effectively and affordably evaluate the impact of utilizing LLHD on nurses’ time allocation at different locations, with a specific focus on patient rooms, we employed an innovative methodology using mobile tracking devices.”

Even less research has been conducted on whether implementing LLHD yields the intended improvements. While studies evaluated the financial performance of Lean Integrated Project Delivery processes (e.g., Tillmann & Echblad, 2023), there is limited evidence regarding the impact of Lean hospital design on the work processes of hospital staff (Boyer et al., 2011; Chbaly, 2022; Hicks et al., 2015). These predominantly focus on the implementation rather than their outcomes. Thus, there is a paucity of research on the effectiveness of applying this design approach. In the view of CareFac’s top management, this boils down to whether the project resulted in increased time spent by nurses at the patient’s bedside and reduced time spent on nonvalue-adding activities.

To assess whether design principles achieve their intended effects, postoccupancy evaluations (POE) are commonly used (Ding, 2016; Kalantari & Snell, 2017; Mahmood & Tayib, 2021). POE involves evaluating buildings through data collection after they have been occupied for some time (Mahmood & Tayib, 2021). These evaluations may examine both physical and psychosocial aspects (Elf et al., 2017).

Researchers in architecture increasingly emphasize the interplay between healthcare facility design and therapeutic patient outcomes, yet the impact on staff has often been overlooked (Elf et al., 2017). One potential explanation for this neglect is the lack of suitable and cost-effective methods for measuring these effects (Kalantari & Snell, 2017). Furthermore, the activities of healthcare staff can be challenging to measure and collect (Griffiths et al., 2018). Even in cases where experimental designs can be utilized to assess performance improvements, attributing causality tends to be problematic (Walshe, 2007).

Addressing the relationship between staff and the building, this research reports on the results obtained using an affordable mobile tracking device to ascertain whether shorter travel distances by nurses in a hospital correlate with increased time spent with patients, defined by CareFac as “value-added time.” As LLHD informed CareFac’s facility’s design, our research question was: How can we effectively and affordably evaluate the effect of using LLHD on the time nurses spend at different locations, particularly in patient rooms?

***How can we effectively and affordably evaluate the effect of using LLHD on the time nurses spend at different locations, particularly in patient rooms?***

## Method

To address our research question, we conducted an indicative POE following the approach suggested by Preiser (1995). Our study introduced an innovative and cost-effective alternative to traditional observational studies to examine the influence of the physical environment on nurses’ time allocation within a surgical ward. Specifically, we utilized locational data to assess the time nurses spent in different areas, using these data to infer their associated activities, such as walking in corridors, delivering patient care in patient rooms, and handling medication in the pharmacy.

Our study specifically focused on assessing the impact of LLHD on the value-adding time of nursing staff. We followed the hospital’s formal interpretation, defining the time spent by nurses in patient rooms as a critical indicator of value-adding time. Value creation includes activities that collectively meet patients’ needs and expectations, including interpersonal interactions and patient–caregiver engagement (Naidu, 2009; Porter, 2010). Direct communication between nurses and patients, ensuring patients feel heard and understood, plays a pivotal role in building and sustaining these relationships, ultimately resulting in value creation (Kieft et al., 2014).

CareFac aimed to eliminate waste through LLHD facility design, although the concept of waste was challenging to define. Graban (2011) characterizes waste “needless hassle.” Given that a significant portion of waste in nurses’ work processes leads to increased time spent in corridors (e.g., searching for equipment, gathering materials, and dealing with an illogical ward layout), CareFac sought to reduce nurses’ corridor time. In hospital settings, wasted motion often presents itself as unnecessary walking (Graban, 2011). Furthermore, nurses frequently grapple with inefficient processes (Graban, 2011), prompting CareFac to apply LLHD principles to minimize nurses’ time spent in the pharmacy, storage rooms, and utility rooms.

### Setting

**Figure 1.**
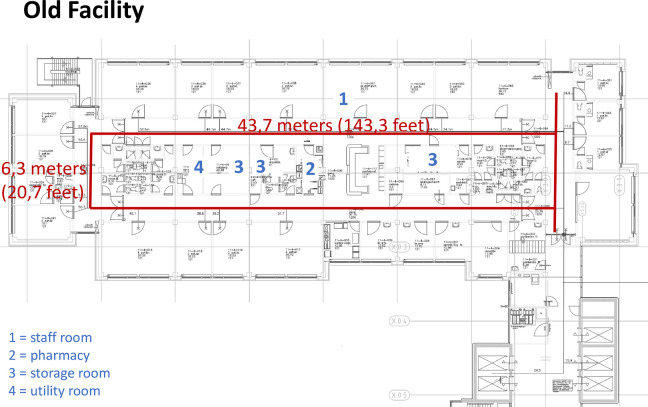
Lay out of the old facility.

**Figure 2.**
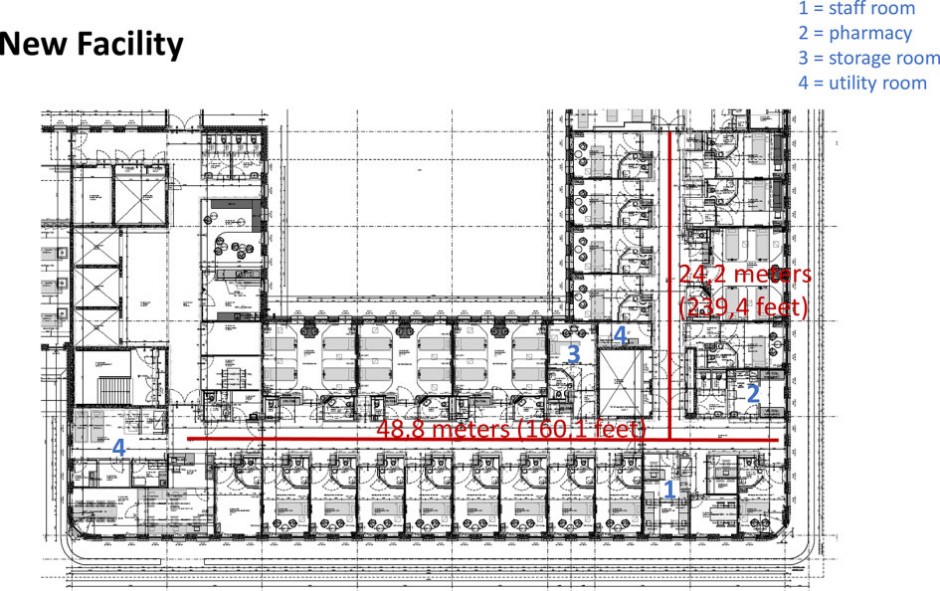
Lay out of the new facility.

On January 28, 2017, CareFac relocated to a new facility designed based on the LLHD framework developed by Grunden and Hagood (2012). The primary objective of the Lean-based design for the new facility was to increase the time nurses spent at patients’ bedsides by reducing “waste time,” for instance, by shortening walking distances. Our study was conducted on ward “4 North,” treating surgical, urology, and orthopedic patients. This ward comprises 32 beds, and the norm for nursing staff is 29 full-time employees. Data were collected from the same nursing team in both the old and new facilities. This specific ward and team were selected as they had already adapted to the new facility’s situation before moving in, thus mitigating confounding factors. Notably, the new facility featured more single-patient rooms compared to the old facility (50% vs. 37.5%). Figures 1 and 2 display the floor plans in the old and new building, respectively.

As noted, CareFac was chosen for this case study due to its unique implementation process of LLHD and the first author’s active role as a participatory action researcher since October 2016. The advantage of a participative action researcher lies in the ability to collect relevant data and gain a deeper understanding of the setting for interpretation (Blake, 2007; Vindrola-Padros et al., 2017). In her role as a formal team member, the first author had privileged access to the complex process of facility development and the moving-in process. She was instrumental in convincing all relevant stakeholders, including the board of directors, senior management of the ward, and staff, to conduct this evaluation. Professional liability concerns often deter stakeholders from evaluating projects, such as the construction of a new facility, out of fear of potential negative feedback (Hadjri & Crozier, 2009).

### Data Collection

CareFac aimed to evaluate the impact of LLHD on the value-adding time of nurses by comparing measurements in the old facility (baseline) to those in the new facility. A controlled study comparing the use of mobile tracking devices to traditional data collection methods was not conducted due to the fixed date of the hospital’s move to the new facility, which left a limited time frame for study setup and data collection in the old facility.

Data collection occurred during two periods: (1) in the old facility from December 19, 2016, to January 4, 2017, and (2) in the new facility from November 22, 2017, to December 8, 2017. Data were collected approximately ten months after the occupation of the new facility on January 28, 2017. During data collection, nurses on the surgical ward were requested to voluntarily carry a mobile tracking device resembling a cell phone in their pockets during their shifts. The tracking devices used were small Android mobile phones (Alcatel one touch Pixi3). Seven devices were available for the day shift, five for evening shifts, and three for night shifts. The total out-of-pocket costs for equipment and licenses were €5,100.

To pinpoint the location of the mobile tracking devices, we employed the Accuware indoors app for Android in conjunction with iBeacons (Estimote location beacons). Fifteen iBeacons were placed in the old building and 11 in the new building at various locations on the ward in addition to Wi-Fi. The app was trained, or “fingerprinted,” by scanning ambient signals to create a dataset. Augmented by the device’s internal sensors, this dataset enabled the estimation of the device’s position and, by extension, the location of the nurse carrying it. This allowed for the systematic and frequent recording of the nurse’s position. Based on the ward’s floor plan, recorded coordinates were linked to specific spaces, enabling the monitoring of the duration of stays within single rooms and movements between rooms. Data were only collected while the mobile tracking device remained on the ward. If a nurse left the ward with the device still in their pocket, data collection ceased. Detailed information about the equipment and software used can be obtained from the first author.

### Privacy

All nurses on the specific team were informed about the project through an email from their team leader based on information provided by the first author. The team leader also verbally informed the nurses during their daily huddle at the project’s outset. It was emphasized that participation was entirely voluntary and anonymous, as it was the nurses’ choice whether to carry a tracker or not. Nurses were informed about the data that would be recorded (the location of their tracker every minute) and the research purpose. Tracking devices were made available in the staff lounge, and nurses were invited by their team leader to take one at the start of their shift if they wished to participate. The act of taking a tracker was considered informed consent, as it required a direct action on the part of the nurses. The investigators had no knowledge of which nurse participated or which tracker they used, as they were not present during data collection and did not possess the names of the team’s nurses. No personal information about team members or patients was collected. The recorded data from the trackers only consisted of tracker ID, coordinates, dates, and times. As a result, the hospital board deemed specific approval from the hospital’s ethics committee unnecessary.

**Figure 3.**
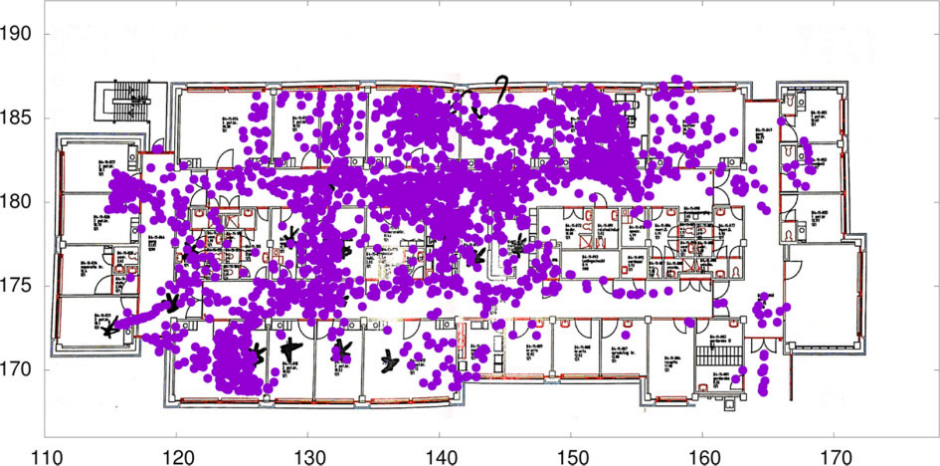
Example of collected and presented data.

Following both data collection periods, heat plots of the raw data were discussed with the team during one of their regular meetings. This provided an opportunity for the nurses to validate findings and assist the investigators in accurately interpreting the data. See Figure 3 for an example of these heat plots.

### Data Processing

The data were further prepared for analysis. Considering that nurses have been found to make a new movement approximately every 67 s (Nazarian et al., 2018), minutes were chosen as the level of analysis (Hendrich et al., 2009; Yen et al., 2018). Since the mobile device recorded its position every minute, each data point represented 1 min. Hours in which the mobile device was on the charger were excluded from the analysis. Additionally, not all spaces on the ward were included in the analysis, for instance, technical rooms and employees’ toilets were excluded as per the nursing staff’s request. Shifts with fewer than 30 data points per hour for over two hr were eliminated to avoid representation issues. Because each mobile tracking device was assigned to a specific shift (day, evening, or night), inter-shift differences were easily analyzed. Finally, each data point was assigned to one of the following spaces based on its coordinates: patient room, corridor, staff room, pharmacy, storage room, or utility room.

**Figure 4.**
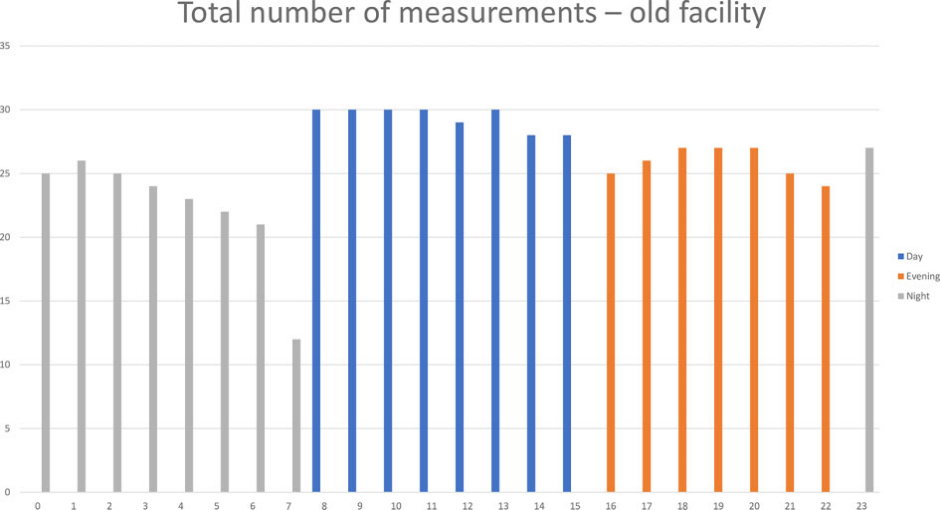
Total number of measurements in the old facility.

**Figure 5.**
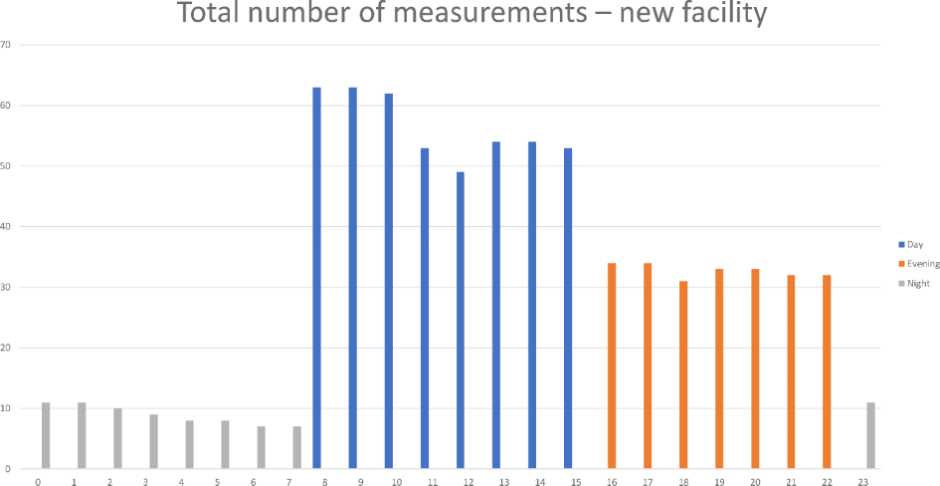
Total number of measurements in the new facility.

Based on these considerations, a dataset was constructed, consisting of 621 working hours of data in the old facility and 762 working hours of data in the new facility (see Figures 4 and 5). Given that multiple tracking devices were available during each shift, data were collected from different nurses during the same hour. However, the number of night shift measurements in the evaluation in the new facility was limited.

### Data Analysis

**Figure 6.**
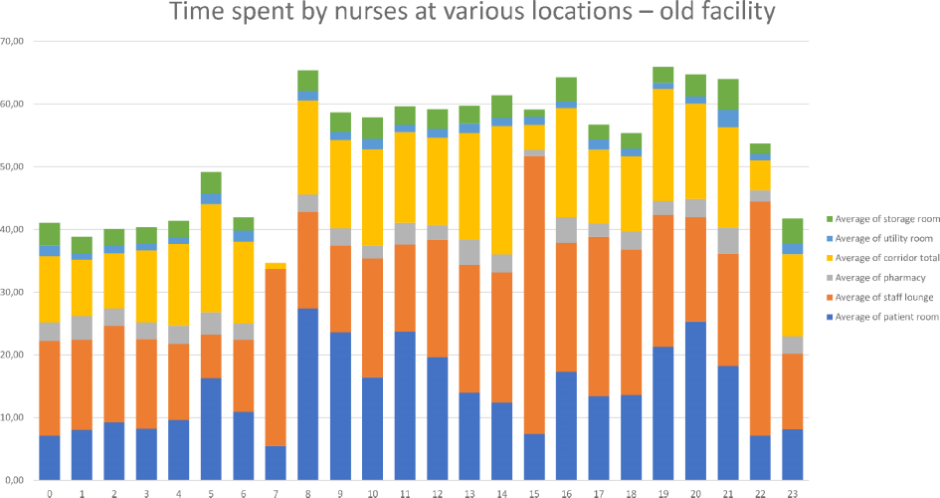
Overview of the time spent by nurses in the old facility.

**Figure 7.**
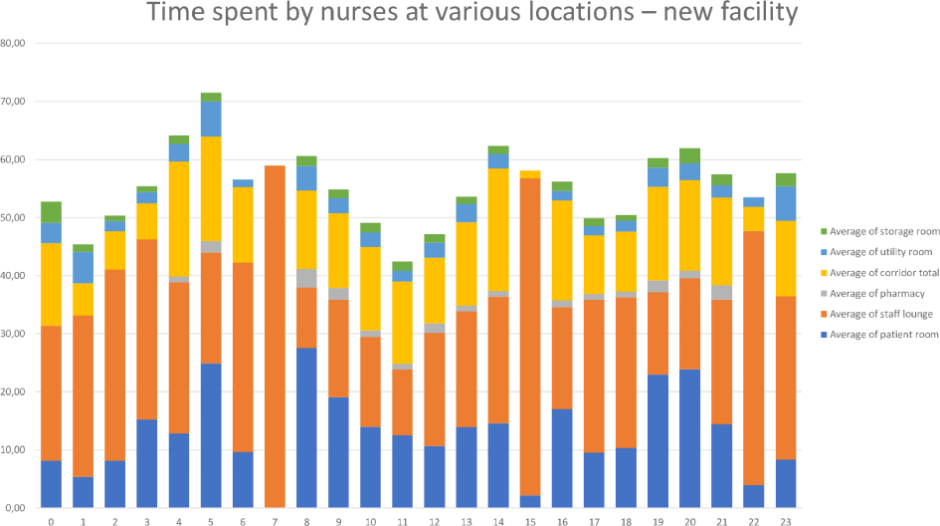
Overview of the time spent by nurses in the new facility.

The collected data were input into Excel, and data visualization was performed using pivot tables and graphs. Graphical summaries were created to display the time nurses spent in patient rooms, corridors, the pharmacy rooms, the storage room, the staff room, and the utility room both before and after moving into of the new facility (see Figures 6 and 7).

The tracking devices recorded an average of 50.11 min per hour per device (nurse). Missing minutes per hour could be attributed to nurses leaving the ward for various reasons, such as transporting patients to operating rooms, taking breaks in the hospital canteen, or accessing their lockers on a different floor. Additionally, technical issues, such as a loss of Wi-Fi signal or depleted device batteries, occasionally resulted in missed measurements. In addition to graphical comparisons, two-sample *t*-tests were conducted to identify differences in the time spent by nurses at various locations.

## Results

A systematic comparison of the time spent in specific rooms before and after moving into the new facility (see Table 1) revealed no statistically significant difference in the time spent by nurses in patient rooms or corridors in the old versus the new facility. This indicates that there were no significant changes in the value-adding time of nurses or the time spent in corridors between the old and the new facilities designed based on LLHD principles.

Further analysis of tracker data, when comparing the old building to the new building, revealed a notable decrease in the amount of time nursing staff spent in the department’s pharmacy and storage room after moving-in, signifying a reduction in what is classified as “waste time.” The reduced time in the pharmacy can be attributed to the new facility’s pharmacy design, which was aligned with Lean principles to minimize the time required for locating medications and to separate storage space from the area dedicated to medication preparation. This separation reduced distractions during medication preparation. Additionally, the new facility introduced an innovative logistic supply system with bandage carts placed at each patient room, which likely contributed to the decreased time spent in storage rooms.

Conversely, the data revealed that nurses spent significantly more time in the staff lounge during their shifts in the new facility compared to the old one. Moreover, nurses also spent significantly more time in the utility room, although the overall amount of time spent there was limited. This increase in utility room time might be linked to the implementation of a new waste disposal system in the new facility, which may have experienced initial challenges (see Table 1).

**Table 1.** Differences for the Mean Before Versus After Occupancy.

Location	Mean “Before Occupancy Time” (Minutes Spend per Hour per Nurse)	Standard Deviation “Preoccupancy Time”	Mean “After Occupancy Time” (Minutes Spend per Hour per Nurse)	Standard Deviation “Postoccupancy Time”	*p* Value for Difference of Median Pre-and Postoccupancy
Patient room	14.92	10.70	15.32	11.59	0.538
Corridors	13.47	8.36	13.52	7.98	0.914
Staff lounge	19.41	16.90	23.24	19.78	**0.000**
Pharmacy	2.94	2.19	1.54	1.34	**0.000**
Utility room	1.46	0.76	2.71	2.77	**0.000**
Storage room	3.06	2.59	1.64	1.12	**0.000**

*Note*. *p* < .05 is shown bold.

It is important to note that, despite the statistical significance of the differences in the minutes nurses spent at specific locations in the old facility compared to the new LLHD facility, the absolute differences were relatively small. The total average reduction in process time amounted to approximately 1.5 min per hour per nurse, equivalent to a 2.5% time-saving. This reduction did not result in an increase in value-adding time, as defined by CareFac.

Additionally, our data revealed a considerable variation in the time nurses spent at different locations, both in the old and the new facilities (indicated by the standard deviations in Table 1). This variation could be associated with differences in how nurses allocated their time on weekdays versus weekends. Other potential explanations include differences in bed occupancy, variations in patient conditions, and other unobserved factors. Unfortunately, these data were not collected due to patient privacy regulations, combined with the need to complete measurements before the relocation from the old facility.

## Discussion

The focal hospital commissioned a study to measure the impact of the new facility on the allocation of nurses’ time in different locations, particularly within patient rooms. We pioneered an innovative method to do so.

Previous pre–post evaluations have adopted diverse instruments to collect data on staff experiences: self-reported experiences and pedometer data (Maben et al., 2016), shadowing and questionnaires (Kevdzija & Marquardt, 2022), and surveys and interviews (Kalantari & Snell, 2017). These methods are often time-consuming, expensive—one of the barriers for conducting POEs (Kalantari & Snell, 2017)—and rely on subjective estimates.

In our study, we opted for a more objective methodology to collect data, seeking to gain deeper insights into the various activities nurses engage in. Employing this approach, we observed that using LLHD seemed to affect how nurses distributed their time across different locations. Nevertheless, ascertaining precisely how LLHD influenced nurses’ time allocation remained a challenging task. On the one hand, our findings indicated no significant difference in the time nurses spent in patient rooms after the transition to the new facility when compared to the previous facility, implying that the new facility did not enhance nurses’ value-adding time. On the other hand, our results showed a shift in activity allocation, with a notable reduction in the time nurses dedicated to activities classified as “waste time,” such as locating or collecting materials and equipment in the storage room (Westbrook et al., 2011). Given that the new facility featured 60% more single-patient rooms, it was expected that there would be an increase in walking distances and time spent in corridors, which is a common disadvantage associated with single-patient rooms according to prior research (e.g., Donetto et al., 2017; Maben et al., 2016; Søndergaard et al., 2022). Surprisingly, we found that the time spent in corridors did not significantly change.

While our POE approach provided insights into the reduction of “waste time,” it did not conclusively demonstrate whether the saved time was redirected to value-added activities, specifically at patients’ bedsides. Nonetheless, we did observe another noteworthy shift in activity allocation, as evidenced by the increased time nurses spent in the staff lounge. Activities conducted in the staff lounge varied, encompassing informal communication during breaks, administrative tasks, and formal meetings. This increase could imply higher inter-professional interactions (cf. Gray et al., 2015), which have been associated with improved patient care and staff satisfaction (Westbrook et al., 2011). As nurses engage in both nonproductive and value-adding activities within the staff lounge, this finding is inconclusive.

### Recommendations for Future Research

As noted in the introduction, our study utilized an affordable and rapid method to conduct a POE in response to our research question concerning the impact of LLHD on nurses’ time allocation in various locations, particularly in patient rooms, and how this evaluation can be performed in a cost-effective and efficient manner. We align with the senior management at CareFac and several scholars who advocated for the validation and confirmation of results related to Lean implementations in healthcare on a larger scale (e.g., D’Andreamatteo et al., 2015; Filser et al., 2017; Hicks et al., 2015).

Despite the valuable insights that POEs provide into the effectiveness of healthcare design, they have not yet become a common practice in the development of new healthcare facilities as highlighted by Kalantari and Snell (2017). Conducting POEs in new hospital facilities presents several challenges. Firstly, the process of conducting a POE can be time-consuming and resource-intensive, especially when it involves the registration of all activities across various spaces as noted by Hadjri and Crozier (2009). Secondly, prior POEs often relied on subjective, self-reported user experiences, which limited the generalizability of the results beyond the specific research context as mentioned by Li et al. (2018). Consequently, there is a pressing need for cost-effective research methods that can objectively measure the intricate impact of specific design features on patient outcomes and staff satisfaction (Hadjri & Crozier, 2009).

The mobile tracking devices employed in our study offer a cost-effective solution requiring minimal time and generating objective measurements. However, these devices should be complemented with other data sources in a POE to provide a more comprehensive understanding of newly adopted design methods like LLHD. We recommend a mixed-method approach that combines quantitative sensor data with qualitative methods, such as shadowing. This approach can help assess whether quantitative differences are directly attributable to the design or influenced by contextual variables as suggested by Benders, Bal, and Vermeerbergen (2019b). Factors such as patient characteristics and logistic information may explain variations in nurses’ time-use patterns. Given the significant variation we observed in nurses’ activity patterns, supplementary qualitative data are essential for interpreting the objective time data.

Like many pioneering research methods, our data collection and analysis processes encountered challenges (Lima et al., 2021). One of these challenges involved assessing the precision and accuracy of the tracking devices, as this innovative method had not yet been fully calibrated. To further validate this activity tracking approach, we recommend that future studies also employ this methodology in preoccupancy evaluation/POE. Given the rapid pace of technological advancements, more precise devices and detailed software will likely be available, facilitating more accurate measurements of nurses’ time allocation.

Finally, we advise not only measuring the time spent by hospital staff in various locations but also using software to create spaghetti diagrams that map nurses’ travel paths. This additional approach can help evaluate expected travel paths and distances compared to the actual paths.

While the first author played a role as a participatory action researcher (Blake, 2007) and was embedded in the hospital organization, it is important to note that she was not a nurse and was not integrated into the analyzed ward. Active involvement of nurses in evaluative research can enhance the understanding and interpretation of data analysis and improve clinical practice environments as demonstrated by Cusack et al. (2018). In line with the call made by Hadjri and Crozier (2009) for further research on the relationship between new facilities and an increase in staff value, we encourage other scholars to build upon our findings and explore this intriguing area.

## Conclusion

In our pursuit to assess how we can effectively and affordably evaluate the effect of using LLHD on the time nurses’ time spent at different locations, particularly in patient rooms, we drew on an innovative methodology using mobile tracking devices.

This approach proved to be both suitable and cost-effective for gathering data regarding the time nurses allocated to specific locations. We utilized these data to determine whether this time was dedicated to value-adding activities or should be categorized as “waste time.”

Our analysis shows that in this case, the use of LLHD as a guiding concept to design a new hospital facility did not result in nurses spending more time in patient rooms. Judged by this proxy, using LLHD appears not lead to more value-adding time.

However, we did observe four statistically significant changes in time spent at various locations: more time in the staff lounge and utility room, and less in the pharmacy and storage rooms. Interpreting these changes in terms of value-adding versus waste time requires in the first place an adequate operationalization of these constructs and, secondly, in line therewith, supplementary qualitative research to gather appropriate data.

## Implications for Practice

- Utilizing mobile tracking devices provides a suitable and cost-effective method for obtaining objective measurements to assess the whereabouts of hospital staff. This, in turn, can be valuable for evaluating design concepts.
- The interpretation of tracker data necessitates additional qualitative information.
- Streamlining time-consuming activities among nurses in a newly designed healthcare facility based on LLHD doesn’t inherently translate to an increased allocation of time for value-adding tasks.

## References

[bibr3-19375867231226440] Benders J. Van Grinsven M. Ingvaldsen J. (2019a). The persistence of management ideas: How framing keeps lean moving. In Sturdy A. Heusinkveld S. Reay T. Strang D. (eds.), The Oxford handbook of management ideas (pp. 271–285). Oxford University Press.

[bibr2-19375867231226440] Benders J. Bal M. Vermeerbergen L. (2019b). Structure please: Continuous improvement and employee consequences in a dynamic task environment. Sustainability, 11(20), 55–64.

[bibr4-19375867231226440] Blake M. K. (2007). Formality and friendship: Research ethics review and participatory action research. ACME: An International Journal for Critical Geographies, 6(3), 411–421.

[bibr5-19375867231226440] Boyer M. Brandenburg L. Wellman J. (2011). Integrated facility design at Seattle Children’s Hospital. In Wellman J. Hagan P. Jeffries H. (eds.), Leading the healthcare journey. Driving culture change into increase value (pp. 215–234). Productivity Press.

[bibr6-19375867231226440] Chbaly H. (2022). The challenges and advantages of implementing a lean-led design approach. Architecture, 2(1), 157–174. 10.3390/architecture2010009

[bibr7-19375867231226440] Cusack C. Cohen B. Mignone J. Chartier M. J. Lutfiyya Z. (2018). Participatory action as a research method with public health nurses. Journal of Advanced Nursing, 74(7), 1544–1553. 29489024 10.1111/jan.13555

[bibr8-19375867231226440] D’Andreamatteo A. Ianni L. Lega F. Sargiacomo M. (2015). Lean in healthcare: A comprehensive review. Health Policy, 119(9), 1197–1209. 10.1016/j.healthpol.2015.02.002 25737260

[bibr9-19375867231226440] Ding S. (2016). Evidence-based design utilized in hospital architecture and changing the design process: A hospital case study. University of Missouri–Columbia.

[bibr10-19375867231226440] Donetto S. Penfold C. Anderson J. Robert G. Maben J. (2017). Nursing work and sensory experiences of hospital design: A before and after qualitative study following a move to all-single room inpatient accommodation. Health and Place, 46, 121–129. 10.1016/j.healthplace.2017.05.001 28527327 PMC5533937

[bibr11-19375867231226440] Elf M. Nordin S. Wijk H. Mckee K. J. (2017). A systematic review of the psychometric properties of instruments for assessing the quality of the physical environment in healthcare. Journal of Advanced Nursing, 73(12), 2796–2816. 10.1111/jan.13281 28207946

[bibr12-19375867231226440] Filser L. D. da Silva F. F. de Oliveira O. J. (2017). State of research and future research tendencies in lean healthcare: A bibliometric analysis. Scientometrics, 112(2), 799–816. 10.1007/s11192-017-2409-8

[bibr13-19375867231226440] Graban M. (2011). Lean hospitals: Improving quality, patient safety and employee statisfaction. CRC Press.

[bibr14-19375867231226440] Gray C. S. Wilkinson A. Alvaro C. Wilkinson K. Harvey M. (2015). Building resilience and organizational readiness during healthcare facility redevelopment transitions: Is it possible to thrive? Health Environments Research & Design Journal, 9(1), 10–33. 10.1177/1937586715593552 26205401

[bibr15-19375867231226440] Griffiths P. Recio-Saucedo A. Dall’Ora C. Briggs J. Maruotti A. Meredith P. Smith G. B. Ball J. (2018). The association between nurse staffing and omissions in nursing care: A systematic review. Journal of Advanced Nursing, 74(7), 1474–1487. 10.1111/jan.13564 29517813 PMC6033178

[bibr16-19375867231226440] Grunden N. Hagood C. (2012). Lean-led hospital design, creating the efficient hospital of the future. Productivity Press.

[bibr17-19375867231226440] Hadjri K. Crozier C. (2009). Post-occupancy evaluation: Purpose, benefits and barriers. Facilities, 27(1/2), 21–33. 10.1108/02632770910923063

[bibr18-19375867231226440] Hendrich A. Chow M. P. Bafna S. Choudhary R. Heo Y. Skierczynski B. A. (2009). Unit-related factors that affect nursing time with patients: Spatial analysis of the time and motion study. Health Environments Research & Design Journal, 2(2), 5–20. 21161927 10.1177/193758670900200202

[bibr19-19375867231226440] Hicks C. McGovern T. Prior G. Smith I. (2015). Applying lean principles to the design of healthcare facilities. International Journal of Production Economics, 170(Pt B), 677–686. 10.1016/j.ijpe.2015.05.029

[bibr20-19375867231226440] Kalantari S. Snell R. (2017). Post-occupancy evaluation of a mental healthcare facility based on staff perceptions of design innovations. Health Environments Research & Design Journal, 10(4), 121–135. 10.1177/1937586716687714 28125896

[bibr21-19375867231226440] Kevdzija M. Marquardt G. (2022). Impact of distance on stroke inpatients’ mobility in rehabilitation clinics: A shadowing study. Building Research and Information, 50(1/2), 74–88. 10.1080/09613218.2021.2001302

[bibr22-19375867231226440] Kieft R. A. M. M. De Brouwer B. B. J. M. Francke A. L. Delnoij D. M. J. (2014). How nurses and their work environment affect patient experiences of the quality of care: A qualitative study. BMC Health Services Research, 14(1). 1–10. 10.1186/1472-6963-14-249 24923663 PMC4064111

[bibr23-19375867231226440] Knudsen S. V. Laursen H. V. B. Johnsen S. P. Bartels P. D. Ehlers L. H. Mainz J. (2019). Can quality improvement improve the quality of care? A systematic review of reported effects and methodological rigor in plan-do-study-act projects. BMC Health Services Research, 19(1). 1–10. 10.1186/s12913-019-4482-6 31585540 PMC6778385

[bibr24-19375867231226440] Li P. Froese T. M. Brager G. (2018). Post-occupancy evaluation: State-of-the-art analysis and state-of-the-practice review. Building and Environment, 133, 187–202. 10.1016/j.buildenv.2018.02.024

[bibr25-19375867231226440] Lima R. M. Dinis-Carvalho J. Souza T. A. Vieira E. Gonçalves B. (2021). Implementation of lean in health care environments: An update of systematic reviews. International Journal of Lean Six Sigma, 12(2), 399–431. 10.1108/IJLSS-07-2019-0074

[bibr26-19375867231226440] Maben J. Griffiths P. Penfold C. Simon M. Anderson J. E. Robert G. Pizzo E. Hughes J. Murrells T. Barlow J. (2016). One size fits all? Mixed methods evaluation of the impact of 100% single-room accommodation on staff and patient experience, safety and costs. BMJ Quality & Safety, 25(4), 241–256. 10.1136/bmjqs-2015-004265 PMC4819646 26408568

[bibr27-19375867231226440] Mahmood F. J. Tayib A. Y. (2021). Healing environment correlated with patients’ psychological comfort: Post-occupancy evaluation of general hospitals. Indoor and Built Environment, 30(2), 180–194. 10.1177/1420326X19888005

[bibr28-19375867231226440] Moraros J. Lemstra M. Nwankwo C. (2016). Lean interventions in healthcare: Do they actually work? A systematic literature review. International Journal for Quality in Health Care, 28(2), 150–165. 10.1093/intqhc/mzv123 26811118 PMC4833201

[bibr29-19375867231226440] Naidu A. (2009). Factors affecting patient satisfaction and healthcare quality. International Journal of Health Care Quality Assurance, 22(4), 366–381. 10.1108/09526860910964834 19725209

[bibr30-19375867231226440] Nazarian M. Price A. Demian P. Malekzadeh M. (2018). Design lessons from the analysis of nurse journeys in a hospital ward. Health Environments Research & Design Journal, 11(4), 116–129. 29902936 10.1177/1937586718779244

[bibr31-19375867231226440] Porter M. E. (2010). What is value in health care? New England Journal of Medicine, 363(26), 2477–2481. 21142528 10.1056/NEJMp1011024

[bibr32-19375867231226440] Preiser W. F. E. (1995). Post-occupancy evaluation: How to make buildings work better. Facilities, 13(11), 19–28. 10.1108/02632779510097787

[bibr33-19375867231226440] Schouten H. Heusinkveld S. van der Kam W. Benders J. (2021). Implementing lean-led hospital design; lessons gained at a pioneer. Journal of Health Organization and Management, 35(1), 1–16. 10.1108/JHOM-08-2019-0250 33047903

[bibr34-19375867231226440] Scoville R. Little K. (2014). Comparing lean and quality improvement: IHI white paper. www.ihi.org

[bibr35-19375867231226440] Shortell S. M. Blodgett J. C. Rundall T. G. Kralovec P. (2018). Use of lean and related transformational performance improvement systems in hospitals in the United States: Results from a national survey. Joint Commission Journal on Quality and Patient Safety, 44(10), 574–582. 10.1016/j.jcjq.2018.03.002 30243359

[bibr36-19375867231226440] Søndergaard S. F. Beedholm K. Kolbæk R. Frederiksen K. (2022). Patients’ and nurses’ experiences of all single-room hospital accommodation: A scoping review. Health Environments Research & Design Journal, 15(1), 292–314. 10.1177/19375867211047548 34636692

[bibr37-19375867231226440] Tillmann P. A. Eckblad S. (2023). Managing human-centered innovation within TVD in healthcare project [Conference session]. Proceedings of the 31st Annual Conference of the International Group for Lean Construction (IGLC31), Lille, France (pp. 1082–1091). 10.24928/2023/0190

[bibr38-19375867231226440] Vindrola-Padros C. Pape T. Utley M. Fulop N. J. (2017). The role of embedded research in quality improvement: A narrative review. BMJ Quality and Safety, 26(1), 70–80. 10.1136/bmjqs-2015-004877 PMC5256405 27129492

[bibr39-19375867231226440] Walshe K. (2007). Understanding what works-and why-in quality improvement: The need for theory-driven evaluation. International Journal for Quality in Health Care, 19(2), 57–59. 10.1093/intqhc/mzm004 17337518

[bibr40-19375867231226440] Westbrook J. I. Duffield C. Li L. Creswick N. J. (2011). How much time do nurses have for patients? A longitudinal study quantifying hospital nurses’ patterns of task time distribution and interactions with health professionals. BMC Health Services Research, 11, 1–12. 10.1186/1472-6963-11-319. 22111656 PMC3238335

[bibr41-19375867231226440] Womack J. Jones D. (1996). Lean thinking: Banish waste and create wealth in your corporation. Simon & Schuster.

[bibr42-19375867231226440] Yen P. Y. Kellye M. Lopetegui M. Saha A. Loversidge J. Chipps E. M. Gallagher-Ford L. Buck J. (2018). Nurses’ time allocation and multitasking of nursing activities: A time motion study. AMIA Annual Symposium Proceedings, 2018, 1137–1146. 30815156 PMC6371290

